# Analytical investigation of nonreciprocal response in 1D nonlinear photonic crystals

**DOI:** 10.1038/s41598-017-06771-2

**Published:** 2017-07-26

**Authors:** Ronger Lu, Jiachu Jiang, Ruizhi Zhao, Xia Feng, Xuhao Hong, Chao Zhang, Yiqiang Qin, Yongyuan Zhu

**Affiliations:** 1 National Laboratory of Solid State Microstructures and Collaborative Innovation Center of Advanced Microstructures and Key Laboratory of Modern Acoustics, Nanjing University, Nanjing, 210093 China; 2 College of Engineering and Applied Sciences, Nanjing University, Nanjing, 210093 China; 3 School of Physics, Nanjing University, Nanjing, 210093 China

## Abstract

The nonreciprocal response of the SHG process in 1D periodical nonlinear photonic crystals with a defect embedded has been theoretically studied by solving the nonlinear coupled equations. The nonreciprocal response has been deduced analytically with the solution of non-reciprocity parameters obtained. The result shows that as the non-reciprocity approaches 100%, the crystal length and the input power needed increase at a logarithmic rate. Any target nonreciprocal response can be reached in this structure by adjusting the structure parameters.

## Introduction

Recent years have witnessed a growing tendency to study nonreciprocal structures and devices in many different areas^1–3^. Inspired by the development of photoelectric diode^4, 5^, researchers have successively invented the acoustic diode^6, 7^ and the thermal diode^8, 9^. According to Lorentz reciprocity theorem^10^, the light propagation process is usually reversible. Therefore, it is difficult to achieve non-reciprocity in optical field. However, several effective ways have been figured out to overcome this problem in recent years. For example, a metal-silicon waveguide system was proposed in 2011, which enabled light to propagate unidirectionally on silicon chips by adjusting light potential energy^11^. In nonlinear optics, an all-optical diode made of a defect embedded in lithium niobate channel waveguide was numerically demonstrated with a spatially nonreciprocal response^12, 13^. External electric field can be employed to further improve the isolating performance for this structure^14–16^, and multiple quasi-phase-matching (QPM) technique was also introduced to realize all-optical isolating action^17^.

In these researches, numerical computation is the main approach used to study the nonreciprocal process, which may not directly explain why it shows a nonreciprocal response as well as how to get a bigger response. In this paper, we have studied the above 1D defective structure theoretically and obtained a series of analytical solutions not only for the second-harmonic output but also for the nonreciprocal response. Further derivations have been implemented and some new results have been revealed from the solutions. The role of the non-reciprocity parameter *P* as well as its corresponding realization conditions has been discussed in detail.

## SHG output solutions and simulations for forward and backward processes

**Figure 1:**
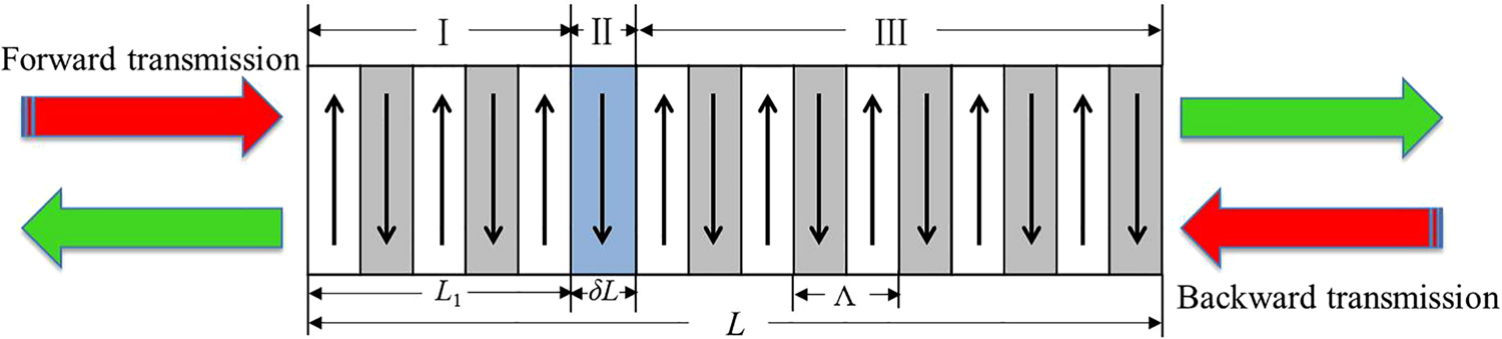
A schematic of the forward and backward SHG processes in a 1D nonlinear photonic crystal with a defect embedded.

It is well known that the field distributions of the SHG process in homogenous nonlinear crystals can be solved exactly with the help of Jacobi elliptic functions^18^. In this paper we show that the nonreciprocal SHG process in 1D defective nonlinear photonics crystal (NPC) structures can be solved in a similar manner. The schematic of the defective NPC structure we studied is shown in Fig. 1, where the up and down arrows represent the domain poling directions. Supposing the whole length of the NPC is *L*, a defect with a width of *δL* is embedded at the position *x* = *L*_1_. Theoretically, the whole structure can be treated as three sections, where the first and third sections have the same period (denoted as Λ) but different initial conditions and the second section is the embedded defect inducing phase shift between the fundamental wave (FW) and second-harmonic wave (SHW). The nonreciprocal responses of the structure can be revealed by studying the forward and backward SHG processes separately, which can be treated analytically with the nonlinear coupled equations.

Firstly, we focus on the forward SHG process. The nonlinear coupled equations for this process are usually expressed as^19^:

1 $$\{\begin{array}{rcl}\frac{d{A}_{1}}{dx} & = & -iKf(x){A}_{2}{A}_{1}^{\ast }\exp (-i{\rm{\Delta }}kx)\\ \frac{d{A}_{2}}{dx} & = & -\frac{1}{2}iKf(x){A}_{1}^{2}\exp (i{\rm{\Delta }}kx)\end{array}$$

where *A*_1_, *A*_2_ refer to the field distributions of FW and SHW, respectively. *K* is the nonlinear coupling coefficient and *f*(*x*) represents the structure function of the NPC. Δ*k* = *k*_2_ − 2*k*_1_ describes the phase mismatching in the SHG process, where *k*_1_ and *k*_2_ are respectively the wave vectors of FW and SHW.

For the first section of the NPC (0 ~ *L*_1_), when the QPM condition is satisfied^20–22^, Eq. (1) can be simplified as:

2 $$\{\begin{array}{rcl}\frac{d{A}_{1}}{dx} & = & -i{K}_{1}{A}_{2}{A}_{1}^{\ast }\\ \frac{d{A}_{2}}{dx} & = & -\frac{1}{2}i{K}_{1}{A}_{1}^{2}\end{array}$$

where *K*_1_ = *K* · *g*_*m*_, and *g*_*m*_ is the Fourier coefficient of the reciprocal vector used in the QPM process. Eq. (2) can be solved easily with the initial conditions of *A*_1_(0) = *A*_0_, *A*_2_(0) = 0, and the following solutions for *A*_1_ and *A*_2_ at the position *x* = *L*_1_ can be obtained^17^:

3 $$\{\begin{array}{rcl}{A}_{1}({L}_{1}) & = & {A}_{0}\,{\rm sech}(\frac{\sqrt{2}}{2}{A}_{0}{K}_{1}{L}_{1})\\ {A}_{2}({L}_{1}) & = & -\frac{\sqrt{2}}{2}i{A}_{0}\,\tanh (\frac{\sqrt{2}}{2}{A}_{0}{K}_{1}{L}_{1})\end{array}$$

For the second section (*L*_1_ ~ *L*_1_ + *δL*), a phase shift (denoted as Δ*φ*) is induced between the FW and SHW, which can be written as Δ*φ* = (2*δL*/Λ − 1)*π*.

For the third section (*L*_1_ + *δL* ~ *L*), the simplified coupled equations under the QPM condition are similar with Eq. (2) except additional terms exp(*i*Δ*φ*) and exp(−*i*Δ*φ*) resulting from the second section, which can be expressed as:

4 $$\{\begin{array}{rcl}\frac{d{A}_{1}}{dx} & = & -i{K}_{1}{A}_{2}{A}_{1}^{\ast }\exp (i{\rm{\Delta }}\phi )\\ \frac{d{A}_{2}}{dx} & = & -\frac{1}{2}i{K}_{1}{A}_{1}^{2}\exp (-i{\rm{\Delta }}\phi )\end{array}$$

In this situation, the initial conditions can be determined by Eq. (3), where not only the FW but also the SHW has a non-zero initial value.

If we concentrate on the variation of amplitudes and phases respectively, Eq. (4) can be further reduced to the following ones:

5a $$\{\begin{array}{rcl}\frac{d{y}_{1}}{dx} & = & -{K}_{1}{y}_{1}{y}_{2}\,\sin \,\theta \\ \frac{d{y}_{2}}{dx} & = & \frac{1}{2}{K}_{1}{y}_{1}^{2}\,\sin \,\theta \end{array}$$

5b $$\{\begin{array}{rcl}\frac{d{\phi }_{1}}{dx} & = & -{K}_{1}{y}_{2}\,\cos \,\theta \\ \frac{d{\phi }_{2}}{dx} & = & -\frac{1}{2}{K}_{1}\frac{{y}_{1}^{2}}{{y}_{2}}\,\cos \,\theta \end{array}$$

where *y*_1_, *y*_2_ represent the amplitudes of the FW and SHW respectively while *φ*_1_, *φ*_2_ represent the corresponding phases and *θ* = 2*φ*_1_ − *φ*_2_ − Δ*φ*.

From the above equations and QPM conditions, two first integrals can be drawn. They are

6 $$\{\begin{array}{rcl}\cos \,\theta \cdot {y}_{1}^{2}{y}_{2} & = & {\rm{\Gamma }}\\ {|{y}_{{\rm{1}}}|}^{{\rm{2}}}+2{|{y}_{{\rm{2}}}|}^{{\rm{2}}} & = & {A}_{0}^{2}\end{array}$$

where Γ is a constant and its value can be obtained by substituting Eq. (3) to the above equation:

7 $${\rm{\Gamma }}=\frac{\sqrt{2}}{2}{A}_{0}^{3}\,\sin \,{\rm{\Delta }}\phi {\rm sech}^{2}(\frac{\sqrt{2}}{2}{A}_{0}{K}_{1}{L}_{1})\tanh (\frac{\sqrt{2}}{2}{A}_{0}{K}_{1}{L}_{1})$$

On the basis of Eqs (6) and (5a) can be further solved as:

8 $${\int }_{{y}_{2}({L}_{1})}^{{y}_{2}(L)}\frac{d({y}_{2}^{2})}{\sqrt{{({A}_{0}^{2}-2{y}_{2}^{2})}^{2}{y}_{2}^{2}-{{\rm{\Gamma }}}^{2}}}=\pm {K}_{1}(L-{L}_{1})$$

Supposing that the term under the square root sign can be written as ${({A}_{0}^{2}-2{y}_{2}^{2})}^{2}{y}_{2}^{2}-{{\rm{\Gamma }}}^{2}=4({y}_{2}^{2}-A)$ $({y}_{2}^{2}-B)({y}_{2}^{2}-C)$, the parameters *A*, *B*, *C* can be expressed as^23, 24^:

9 $$\{\begin{array}{rcl}A & = & \frac{{A}_{0}^{2}}{3}+\frac{{A}_{0}^{2}}{3}\,\cos (\zeta +\frac{2\pi }{3})\\ B & = & \frac{{A}_{0}^{2}}{3}+\frac{{A}_{0}^{2}}{3}\,\cos (\zeta +\frac{4\pi }{3})\\ C & = & \frac{{A}_{0}^{2}}{3}+\frac{{A}_{0}^{2}}{3}\,\cos \,\zeta \end{array}$$

where $\zeta =\arccos (-1+27{{\rm{\Gamma }}}^{2}/{A}_{0}^{6})/3$ and *A* ≤ *B* ≤ *C*. Here *A*, *B*, *C* are all real numbers since $0\le 27{{\rm{\Gamma }}}^{2}/{A}_{0}^{6}\le 2$ is always satisfied and thus obviously *ζ* ∈ [0, *π*/3]. Eq. (8) can be further solved as:

10 $${{y}_{2}}^{2}(L)={a}^{2}{{\rm{sn}}}^{2}[b{K}_{1}(L-{L}_{1})\pm F(\gamma ,\varphi ),\gamma ]+A$$

where $F(\gamma ,\varphi )=F(a/b,\arcsin (\sqrt{{y}_{2}^{2}({L}_{1})-A}/a))$ is the Legendre’s incomplete elliptic integral of the first kind, and *a*, *b* are two real constants which are defined by $a=\sqrt{B-A}$ and $b=\sqrt{C-A}$. sn(*z*,*γ*) represents Jacobi elliptic function with modulus *γ* = *a*/*b*. In Eq. (10), we should choose a “+” sign if *y*_2_ is to increase with increasing *x* and a “−” sign if *y*_2_ is to decrease with increasing *x*.

Secondly, for the backward transmission, the results are similar to the forward ones, thus the amplitude for SHW can be expressed as:

11 $${{y}_{2}}^{2}(0)={a}^{^{\prime} 2}{{\rm{sn}}}^{2}[b^{\prime} {K}_{1}{L}_{1}\pm F(\gamma ^{\prime} ,\varphi ^{\prime} ),\gamma ^{\prime} ]+A^{\prime} $$

where $F(\gamma ^{\prime} ,\varphi ^{\prime} )=F(a^{\prime} /b^{\prime} ,\arcsin (\sqrt{{y}_{2}^{2}(L-{L}_{1})-A^{\prime} }/a^{\prime} ))$. Parameters *a*′, *b*′, *A*′ can be obtained by repeating the above deduction processes with the defect position *L* − *L*_1_ instead of *L*_1_.

**Figure 2:**
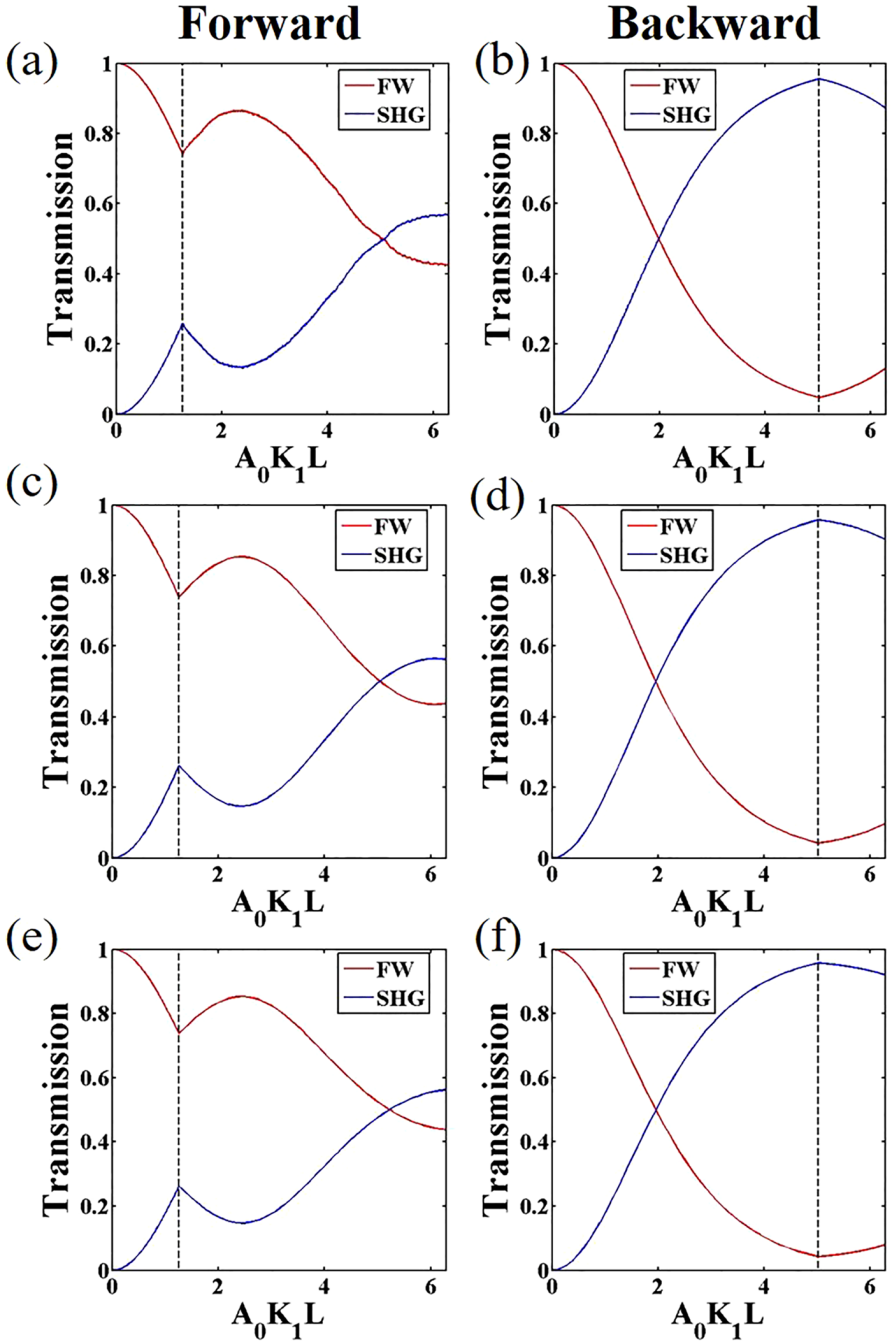
Normalized amplitudes of FW and SHW for the forward and backward processes in the defective NPC. (**a**–**f**) are the corresponding forward and backward propagations calculated on the basis of the original coupled equations, the simplified coupled equations and the analytical solutions, respectively.

Numerical calculations have been conducted together with the above analytical solutions to demonstrate the nonreciprocal response of the defective NPC structure. Assuming that the defect position is *L*_1_ = 0.2*L* and *δL* = 5Λ/6, that is the phase shift Δ*φ* equals to 2*π*/3, the normalized amplitudes of FW and SHW in the NPC are shown in Fig. 2. Figure 2(a) and (b) show the forward and backward transmission processes calculated on the basis of the original coupled equations Eq. (1). Figure 2(c) and (d) are corresponding processes obtained by numerical calculation of the simplified equations Eqs (2) and (4). Whereas Fig. 2(e) and (f) show the corresponding results of the analytical solutions Eqs (10) and (11). We can see that they match well with each other.

## Analytical and numerical derivations of the non-reciprocity parameter

According to [12–15], the non-reciprocity parameter *P* in this SHG process can be defined as:

12 $$P=\frac{|{y}_{2}^{-}|-|{y}_{2}^{+}|}{|{y}_{2}^{-}|+|{y}_{2}^{+}|}$$

Here, superscripts “+” and “−” represent the forward and backward processes, respectively. In the situation that *L* − *L*_1_ ≫ *L*_1_, Eq. (12) can be simplified as:

13 $$P=\frac{1-{\eta }^{+}}{1+{\eta }^{+}}$$

where ${({\eta }^{+})}^{2}=2{|{y}_{2}^{+}|}^{2}/{A}_{0}^{2}$ describes the SHG conversion efficiency of the forward process.

Substituting Eq. (10) into Eq. (13), it is found that when the forward SHG output reaches its minimum, the non-reciprocity achieves the maximum, and vice versa. According to the properties of Jacobi elliptic function, when the forward output achieves the minimum or maximum, the following expressions for the total length of the crystal *L* can be obtained respectively:

14 $$L=\frac{{\rm{1}}}{b{K}_{1}}[nT(\gamma )\pm F(\gamma ,\arcsin \frac{\sqrt{{y}_{2}^{2}({L}_{1})-A}}{a})+b{K}_{1}{L}_{1}]$$

where *T*(*γ*) = *F*(*γ*, *π*/2) describes the period of the first kind of complete elliptic integral and *n* represents positive integers. When *n* is an even number, the forward SHG output reaches the minimum value $\sqrt{A}$ and otherwise achieves the maximum value $\sqrt{B}$. The corresponding extremes of *P* can be solved as

15 $$\{\begin{array}{rcl}{P}_{\min } & = & \frac{\sqrt{3}-2\,\sin (\frac{\zeta }{2}+\frac{\pi }{6})}{\sqrt{3}+2\,\sin (\frac{\zeta }{2}+\frac{\pi }{6})}\\ {P}_{\max } & = & \frac{\sqrt{3}-2\,\sin (\frac{\pi }{6}-\frac{\zeta }{2})}{\sqrt{3}+2\,\sin (\frac{\pi }{6}-\frac{\zeta }{2})}\end{array}$$

It can be analytically solved from Eq. (15) that *P*_max_ equals to *P*_min_ when the condition $27{{\rm{\Gamma }}}^{2}/{A}_{0}^{6}=2$ is satisfied. That means with specific values of *A*_0_ *K*_1_ *L*_1_ and Δ*φ*, no matter what the length of the total crystal, the value of *P* keeps the same.

**Figure 3:**
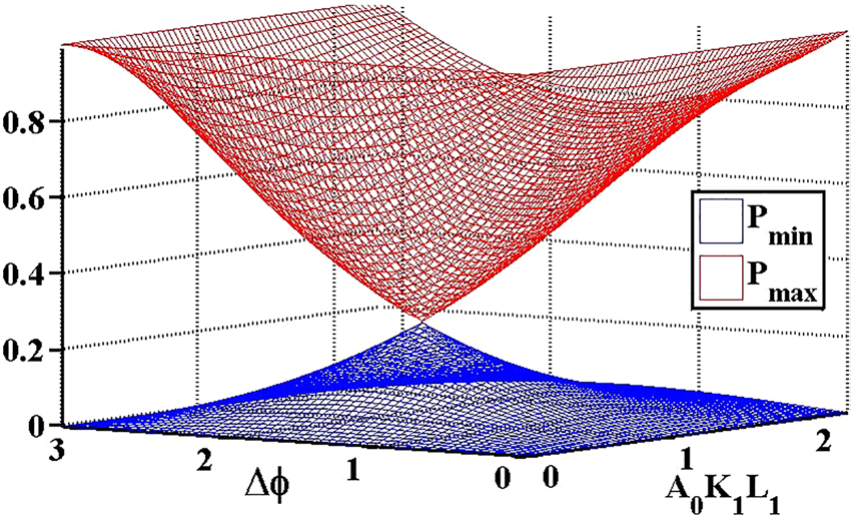
Surface plot of the extremes of the non-reciprocity parameter with different structure parameters.

Surface plot of the extremes of is calculated and shown in Fig. 3. It can be seen that high nonreciprocal responses can be obtained when Δ*φ* is close to 0 or *π*, which corresponds to the situation that Γ is small. Furthermore, if Γ → 0, ideal non-reciprocity can be obtained. The expression of *P*_max_ can be approximately simplified as follows:

16 $${P}_{\max }=1-\frac{2\sqrt{2}{\rm{\Gamma }}}{{A}_{0}^{3}}$$

In this situation, *γ* can be expressed as $\gamma =1-3\sqrt{2}{\rm{\Gamma }}/{A}_{0}^{3}$. Substituting Eq. (16) into Eq. (14), it can be concluded that the total NPC length needed increases logarithmically with *P*_max_ since the corresponding period *T*(*γ*) can be approximately expressed as

17 $$T(\gamma )=\frac{1}{2}\,\mathrm{ln}\,\frac{16}{1-\gamma }$$

**Figure 4:**
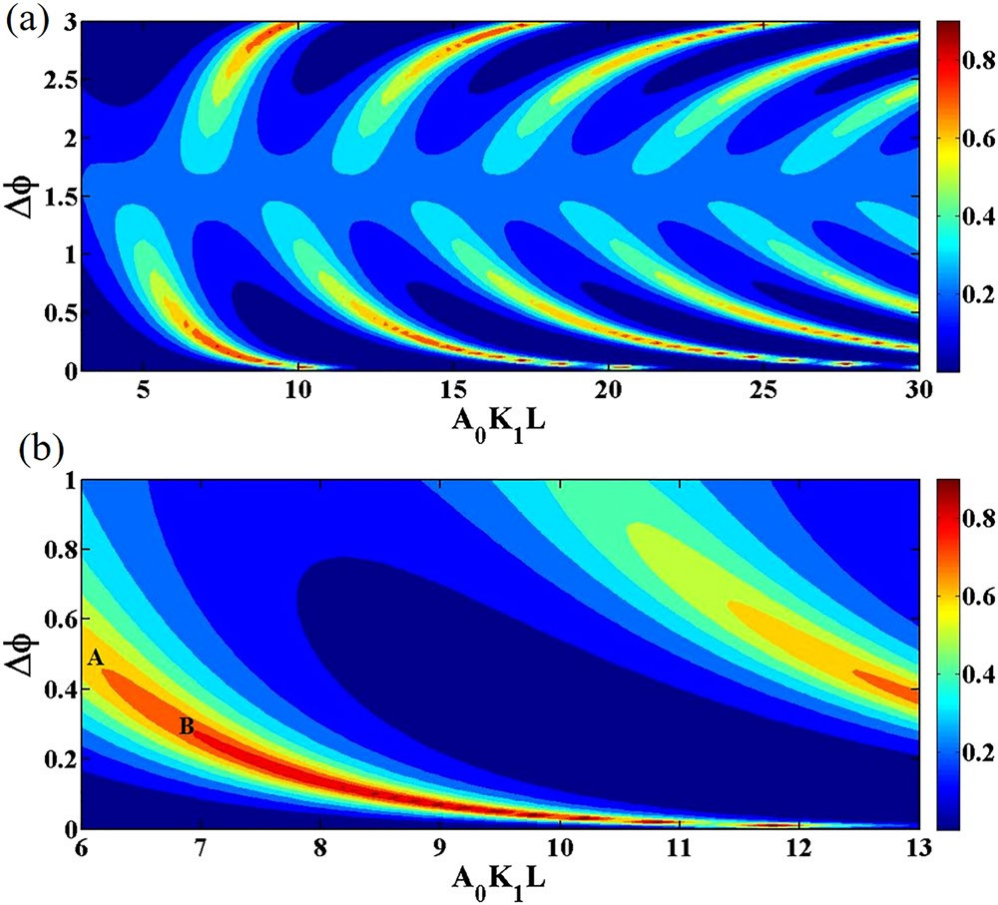
Distribution of non-reciprocity parameter with different values of Δ*φ* under the condition that *A*_0_ *K*_1_ *L*_1_ = 0.3*π*. Different color represents different nonreciprocal responses.

Figure 4 shows the dependence of *P*_max_ and Δ*φ*, where Δ*φ* varies in the range of 0 and *π*, and *A*_0_ *K*_1_ *L*_1_ is set to be 0.3*π*. Figure 4(b) is the partial magnification of Fig. 4(a). It can be seen that the non-reciprocity parameter *P* is periodically distributed for a given Δ*φ*, which is resulted from the periodicity of the Jacobi elliptic function. Besides, when Δ*φ* → 0 or Δ*φ* → *π*, the corresponding period will increase logarithmically, which matches well with the analytical discussions.

Simulation results show that the contrast ratio of the all-optical diode can maintain close to 1. Thus, a near complete nonreciprocal all-optical diode based on a NPC can be realized. As is shown in the Fig. 4(b), the same color describes the same nonreciprocal response. It can be seen that for any target non-reciprocity, a NPC structure with a defect embedded can be designed with specific values of Δ*φ* and *A*_0_ *K*_1_ *L*_1_, which are realized by adjusting the defect width, the crystal length and the FW input. As Δ*φ* moves away from zero, the same nonreciprocal response is kept as long as the total length of the crystal decreases. It means that the target non-reciprocity could be achieved with a shorter crystal length and a lower FW input by adjusting the defect width. For practical applications in a defective NPC structured on the lithium niobate (LN), the nonlinear coefficient *d*_33_ = 27 *pm*/*V*. If a 70% nonreciprocal contrast is required, that is *P*_max_ = 0.7, *A*_0_ *K*_1_ *L* ≈ 6.191 and Δ*φ* ≈ 0.532 can be carried out according to Eqs (7) and (14), sketched by point ‘A’ in Fig. 4(b). The data show that if we set the FW (1064 nm) intensity to be 60 *MW*/*cm* ^2^, the needed total length is 1.3 cm and the required defect width is 3.8 *μm* with the QPM period being 6.6 *μm*. Similarly, if an 80% nonreciprocal contrast is required, the corresponding parameters can be obtained by the same method, shown by point ‘B’ in the figure. Discussions above have explained a universal design of nonlinear crystals. In comparison with previously reported NPC diodes, our results reveal that the design of diodes can be more flexible and easier to control.

## Conclusion

In all, we have theoretically studied the non-reciprocity properties of SHG process realized by a defective 1D NPC. Exact solutions for the SHG output in both forward and backward transmission processes have been derived with elliptic function forms. According to the property of the elliptic function, it can be concluded that for specific values of Δ*φ* and *A*_0_ *K*_1_ *L*_1_, the non-reciprocity parameter has a maximum value as well as a minimum value. It is found that the complete non-reciprocity cannot be realized in this structure but can be approached infinitely. In this case, the crystal length needed increases with the nonreciprocal parameter at a logarithmic rate. Any target non-reciprocity can be obtained by adjusting the defect width, the crystal length and the FW input. These results may lead to a variety of relevant applications, including all-optical diode, optical isolator, amplifiers and so on.

## Methods

It is obvious that there is no non-reciprocity when the defect is at the middle position, which means *L*_1_ = *L*/2. Asymmetric structures should be adopted for significant responses. Hereafter we present a detailed analysis under the condition that *L* − *L*_1_ ≫ *L*_1_. In this situation, it is found that the SHG output in backward transmission process will tend to $\sqrt{2}{A}_{0}/2$ as long as the intensity of the input FW is sufficiently high. Thus Eq. (12) can be simplified as:

18 $$P=\frac{\frac{\sqrt{2}}{2}{A}_{0}-|{y}_{2}^{+}|}{\frac{\sqrt{2}}{2}{A}_{0}+|{y}_{2}^{+}|}$$

Ordering ${({\eta }^{+})}^{2}=2{|{y}_{2}^{+}|}^{2}/{A}_{0}^{2}$ to describe the SHG conversion efficiency of the forward process, Eq. (18) can be written as $P=\frac{1-{\eta }^{+}}{1+{\eta }^{+}}$. As a result, complete reciprocity and non-reciprocity are corresponding to *P* = 0 and *P* = 1, respectively.

Figures for the discussion of the non-reciprocity parameter are all calculated by MATLAB on the basis of the analytical solutions. Figure 3 is obtained with Eq. (15) under the approximate condition that *L* − *L*_1_ ≫ *L*_1_ while Fig. 4 is obtained by accurately substituting the Eqs (10) and (11) into Eq. (12). The digital trends for the corresponding points match well between the two figures, which demonstrated that the approximation above is reasonable.
